# Etiology of Encephalitis in Australia, 1990–2007

**DOI:** 10.3201/eid1509.081540

**Published:** 2009-09

**Authors:** Clare Huppatz, David N. Durrheim, Christopher Levi, Craig Dalton, David Williams, Mark S. Clements, Paul M. Kelly

**Affiliations:** Hunter New England Population Health, Newcastle, New South Wales, Australia (C. Huppatz, D.N. Durrheim, C. Dalton); Australian National University, Canberra, Australian Capital Territory, Australia (M.S. Clements, P.M. Kelly); John Hunter Hospital, New Lambton, New South Wales, Australia (C. Levi, D. Williams)

**Keywords:** Encephalitis, viral encephalitis, infectious encephalitis, viruses, Australia, research

## Abstract

Encephalitis is a clinical syndrome commonly caused by emerging pathogens, which are not under surveillance in Australia. We reviewed rates of hospitalization for patients with encephalitis in Australia’s most populous state, New South Wales, from January 1990 through December 2007. Encephalitis was the primary discharge diagnosis for 5,926 hospital admissions; average annual hospitalization rate was 5.2/100,000 population. The most commonly identified pathogen was herpes simplex virus (n = 763, 12.9%). *Toxoplasma* encephalitis and subacute sclerosing panencephalitis showed notable declines. The average annual encephalitis case-fatality rate (4.6%) and the proportion of patients hospitalized with encephalitis with no identified pathogen (69.8%, range 61.5%–78.7%) were stable during the study period. The nonnotifiable status of encephalitis in Australia and the high proportion of this disease with no known etiology may conceal emergence of novel pathogens. Unexplained encephalitis should be investigated, and encephalitis hospitalizations should be subject to statutory notification in Australia.

## Abstract 2

Unexplained disease etiology in hospitalized patients highlights the importance of surveillance to detect emerging novel pathogens.

Many novel infectious diseases have been reported since 1940; most have been zoonoses originating from wildlife (*1*). Several novel zoonotic viruses, including Hendra virus, Australian bat lyssavirus, and Nipah virus, have resulted in encephalitic illness in humans (*2*–*4*). Australia has witnessed the emergence of several zoonotic and arboviral pathogens associated with encephalitis; these pathogens have been either novel pathogens or pathogens appearing in new geographic locations and include Hendra virus, Murray Valley encephalitis virus, Australian bat lyssavirus, and Kunjin and Japanese encephalitis viruses (*5*–*7*). The appearance of these emerging pathogens that can result in encephalitis raises questions about the etiology of encephalitis in the Australian population and about the adequacy of surveillance for novel pathogens.

Encephalitis is an inflammatory process in the brain parenchyma and is associated with clinical evidence of brain dysfunction (*8*). An infectious etiology of encephalitis is usually suspected in a patient with fever, headache, and signs of diffuse brain dysfunction, often with focal neurologic signs (*9*,*10*). Encephalitis generally results in a serious illness requiring hospitalization. Severe encephalitic illness can lead to death, and survivors frequently experience ongoing neurologic sequelae (*9*,*10*).

Presumably, the most common etiologies of encephalitis are infectious (*11*), and viral pathogens account for most diagnosed cases. In developed countries, the most commonly identified pathogens associated with acute encephalitis are the herpes viruses (*9*,*12*–*15*). Herpes simplex encephalitis is believed to account for 10%–20% of cases (*12*), but pathogen identification is not always possible (*16*).

Several countries have conducted large epidemiologic studies to assess the impact of disease caused by encephalitis and to determine its etiology. Davison et al. reviewed UK hospital records for a 9-year period (1989–1998) and found that the hospitalization rate for viral encephalitis in UK hospitals was 1.5 cases per 100,000 population; 60% of cases were recorded as unidentified viral infection (*17*). During the study period in the United Kingdom, 419 deaths were attributed to viral encephalitis (overall case-fatality rate 6.5/100 cases), but an etiologic organism was identified for only 50% of these deaths (*17*). A smaller prospective study conducted in Finland for a 25-year period (1967–1991) found no etiology for encephalitis in 49% of 322 patients hospitalized with this illness (*18*).

Khetsuriani et al. analyzed national data for the United States over a similar period (1988–1997) to estimate the impact of both viral and nonviral encephalitis hospitalizations (*11*). This study found that the hospitalization rate for encephalitis in the United States was 7.3 per 100,000 population, and no specific etiology was identified in 59.5% of cases. During the study period, a case-fatality rate of 7.4 per 100 cases was recorded. For those persons admitted with encephalitis for which an etiology was identified, most specified a viral etiology (*11*). Also in the United States, the California Encephalitis Study, a large prospective study, found that 63% of hospitalized patients with encephalitis from 1998 through 2005 had encephalitis of unknown etiology, despite extensive laboratory testing (*19*). In another US study of persons whose deaths were associated with encephalitis, 81.5%–86.2% of the deaths resulted from encephalitis with an unknown etiology (*20*).

In Australia, encephalitis, as a syndrome, is not a notifiable disease, and data about trends or even clusters of this disease are not routinely collected. Only laboratory-confirmed encephalitis cases due to certain pathogens (e.g., Murray Valley encephalitis virus, Japanese encephalitis virus, or Australian bat lyssavirus) are notifiable to public health units; thus, the occurrence of encephalitis is not well documented.

A single, small study conducted in Australia’s tropical Northern Territory found that 18 (53%) of 34 encephalitis patients admitted to the Royal Darwin Hospital during a 5-year period (1992–1996) had encephalitis with unexplained etiology (*21*). This level of unknown pathogen etiology suggests that Australia has a similar rate of pathogen identification as that reported in the overseas studies. However, the relative importance of different pathogens may differ because Australia has unique pathogens such as Murray Valley encephalitis virus, Hendra virus, and Australian bat lyssavirus) (*5*).

In this study, we examine the impact of hospitalizations due to encephalitis in New South Wales (NSW), Australia’s most populous state. We also describe trends in pathogen identification in patients hospitalized with encephalitis over the 18-year period 1990–2007.

## Methods

### Data Sources

Data on hospital discharges, deaths, and population for NSW were obtained for 1990–2007 from the Health Outcomes and Information Statistical Toolkit, a collection of databases maintained by the Epidemiology and Surveillance Branch of the NSW Department of Health. The datasets used were from the Inpatient Statistics Collection library. Encephalitis-associated hospital stays were extracted for the period for patients for whom data were complete by using International Classification of Diseases, 9th revision, Clinical Modification codes (ICD-9-CM, Jan 1990–Jun 1998) and International Classification of Diseases, 10th revision, Australian Modification (ICD-10-AM, July 1998–Dec 2007).

Data for encephalitis-associated deaths in NSW were extracted from the Australian Bureau of Statistics death library by using ICD-9 (1990–1997) and ICD-10 (1998–2006) codes. Data for deaths occurring in 2007 were unavailable at the time of extraction. Population statistics from the Australian Bureau of Statistics were accessed through the Health Outcomes and Information Statistical Toolkit for the same period.

### Definitions

An encephalitis-associated hospital stay was defined as a hospitalization for which the primary discharge diagnosis was an ICD-9-CM or ICD-10-AM code for acute encephalitis. Relevant ICD-9-CM and ICD-10-AM codes were obtained by searching the alphabetical list of ICD codes for all codes that include the term enceph, but excluding nonencephalitis conditions (e.g., anencephaly) and including rabies. Additionally, 2 ICD-9-CM codes used for encephalitis-related conditions before July 1998 did not include the prefix enceph-, unlike nomenclatures used in ICD-10-AM encephalitis codes. One of these ICD-9-CM codes was 49.8, defined as other non–arthropod-borne viral diseases of central nervous system –other specified non–arthropod-borne viral diseases of the central nervous system. The other code was 49.9, defined as other non–arthropod-borne viral diseases of central nervous system –unspecified non–arthropod-borne viral diseases of the central nervous system. The use of these codes was found by comparing data extracted for 1998 and 1999. For these 2 years, coding was performed in both ICD-9-CM and ICD-10-AM, and data were then compared to identify the additional codes.

The Table shows the ICD-9-CM and ICD-10-AM encephalitis-associated conditions with codes used for encephalitis hospitalizations during 1990–2007. These codes were further classified by investigators into encephalitis with known pathogens or unknown pathogens (Table).

**Table:** Encephalitis-associated conditions with ICD-9-CM and ICD-10-AM codes most frequently used for primary encephalitis discharge diagnoses, New South Wales, Australia, 1990–2007*

Primary discharge diagnosis ICD code (ICD-9; ICD-10)	No. hospitalizations (% of total)
All hospitalizations	5,926 (100)
Known pathogens	1,800 (30.6)
Herpes viral encephalitis (54.3; B00.4)	763 (12.9)
Varicella encephalitis (52.0; B01.1)	226 (3.8)
*Toxoplasma* meningoencephalitis (130.0; B58.2 )	221(3.7)
Acute disseminated encephalitis (G04.0 in ICD-10)	136 (2.3)
Zoster encephalitis (B02.0 in ICD-10)	105 (1.8)
Subacute sclerosing panencephalitis (46.2; A81.1)	79 (1.3)
Other specified non–arthropod-borne viral diseases of the central nervous system (49.8; A85.8)	71 (1.2)
Enteroviral encephalitis (A85.0 in ICD-10)	49 (0.8)
Listerial meningitis and meningoencephalitis (A32.1 in ICD 10)	28 (0.5)
Encephalitis, myelitis, and encephalomyelitis –postinfectious encephalitis (323.6 in ICD-9)	18 (0.3)
Measles encephalitis (55.0 in ICD 9)	15 (0.3)
Encephalitis, myelitis, and encephalomyelitis –encephalitis in viral diseases classified elsewhere (323.0 in ICD 9)	10 (0.2)
Rubella with neurologic complications –encephalomyelitis due to rubella (56.01 in ICD-9)	8 (0.1)
Meningococcal infection –meningococcal encephalitis (36.1 in ICD-9)	7 (0.1)
Viral encephalitis transmitted by other and unspecified arthropods (64 in ICD-9)	7 (0.1)
Bacterial meningoencephalitis and meningomyelitis, not elsewhere classified (G04.2 in ICD-10)	7 (0.1)
Mumps –mumps encephalitis (72.2 in ICD-9)	6 (0.1)
Other known pathogen codes (24 codes)	44 (0.7)
Unknown pathogens	4,126 (69.6)
Unspecified non–arthropod-borne viral diseases of the central nervous system or unspecified viral encephalitis (49.9; A86)	2,218 (37.4)
Encephalitis, myelitis, and encephalomyelitis –unspecified cause of encephalitis (323.9; G04.9)	1,648 (28.8)
Encephalitis, myelitis, and encephalomyelitis –other cause of encephalitis (323.8; G04.8)	260 (4.4)

*ICD-9-CM, International Classification of Diseases, 9th Revision, Clinical Modification; ICD-10-AM, International Classification of Diseases, 10th Revision, Australian Modification.

### Data Analysis and Ethics

Encephalitis-associated hospitalizations were analyzed by using SAS version 8.0 (SAS Institute, Inc., Cary, NC, USA) by etiologic category, year, age, gender, and hospital location. Hospitalization rates and death rates were calculated by using annualized population estimates from the Australian Bureau of Statistics. Negative binomial regression was used to analyze trends for hospitalization rates of all encephalitis hospitalizations and for those caused by known and unknown pathogens. The regression was repeated with adjustment for age groups over time by using log of the population as an offset and age as a categorical covariate. Trends were represented as annual percentage changes in rates. Ethical clearance was given by Hunter New England Population Health and by the Australian National University Human Research Ethics Committee.

## Results

### Encephalitis Hospitalizations

From January 1990 through December 2007, encephalitis accounted for 5,926 hospitalizations in NSW. The average number of hospitalizations per year was 329 (range 281–393). The most frequently identified etiology of encephalitis was herpes simplex virus infection, which accounted for 763 admissions (12.9%) (Table). Varicella encephalitis (226 admissions or 3.8%) and *Toxoplasma* meningoencephalitis (221 admissions or 3.7%) were also common known etiologies. The etiology of 4,126 admissions (69.6% of total admissions) was unknown.

**Figure 1:**
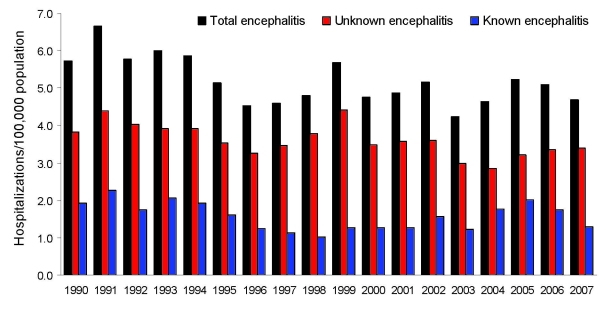
Encephalitis hospitalization rates by year and by known and unknown pathogen etiology, New South Wales, Australia, 1990–2007.

The average annual rate of encephalitis hospitalization was 5.2 per 100,000 population (range 4.2–6.7) (Figure 1). The annual rate for total encephalitis cases was higher for men (average rate 5.7/100,000) than for women (4.7/100,000).

**Figure 2:**
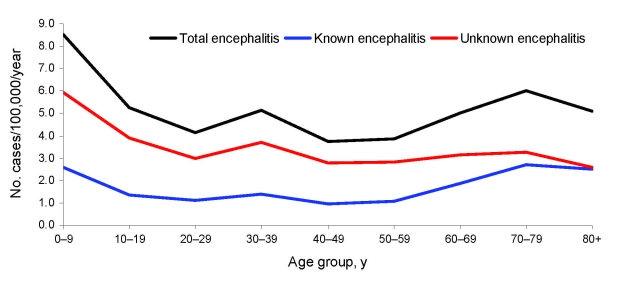
Average rates of encephalitis hospital admissions by 10-year age groups and by known and unknown pathogen etiology, New South Wales, Australia, 1990–2007.

The case-fatality rate for encephalitis during 1990–2006 was 4.6/100 cases. Figure 2 shows the average rate of hospitalization for patients with encephalitis by 10-year age groups during 1990–2007. The highest rates of admission occurred for age groups 0–9 years and >60 years.

### Pathogen Identification

The rate of encephalitis hospitalizations declined for admissions of patients with encephalitis with both known and unknown etiologies (Figure 1). A negative binomial regression model showed a statistically significant decline of 1.4% per year (p = 0.0003, 95% confidence interval [CI] 0.7%–2.2%) for total encephalitis admissions during 1990–2007. This trend was similar for cases of encephalitis with unknown etiologies (decline of 1.4% per year, p = 0.0002, 95% CI 0.7%–2.2%) and for encephalitis cases with known etiologies, although the effect was not statistically significant for pathogens with known etiologies (decline of 1.4% per year, p = 0.13, 95% CI 0.4%–3.2%,). This trend did not change when adjusted for age groups.

The proportion of cases with pathogens of known etiologies was higher for men than for women; the average hospitalization rate for men with encephalitis with a known etiology was 1.9/100,000 compared with 1.3/100,000 for women with encephalitis with a known etiology. In contrast, rates for patients with encephalitis with unknown etiologies were similar for men (3.8/100,000) and women (3.4/100,000). When the diagnosis of *Toxoplasma* encephalitis was excluded, the rate for hospitalizations for men with encephalitis with identified etiologies (1.5/100,000) was similar to the rate for women (1.3/100,000).

The proportion of hospitalizations with encephalitis with known etiology varied little among hospitals; a notable exception was a large Sydney hospital specializing in HIV-related medicine. For this hospital, the higher proportion of patient admissions with encephalitis with known etiology (50%) resulted from a high number of *Toxoplasma* encephalitis admissions.

### Known Causes

**Figure 3:**
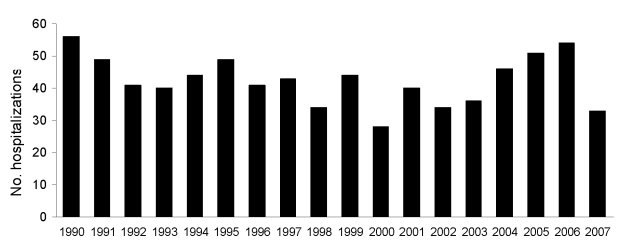
Herpes encephalitis hospitalizations by year, New South Wales, Australia, 1990–2007.

**Figure 4:**
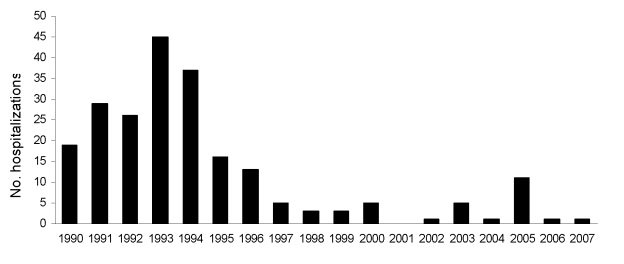
*Toxoplasma* encephalitis hospitalizations by year, New South Wales, Australia, 1990–2007.

**Figure 5:**
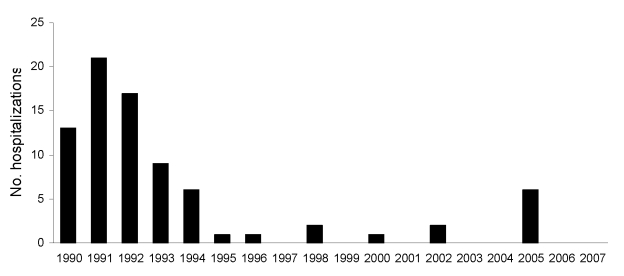
Subacute sclerosing panencephalitis hospitalizations by year, New South Wales, Australia, 1990–2007.

Herpes encephalitis accounted for a relatively stable proportion of encephalitis hospitalizations during the study period (Figure 3). *Toxoplasma* encephalitis hospitalizations increased early in the study period and peaked in 1993 (Figure 4). Few toxoplasmosis hospitalizations occurred during the last 10 years of the study period. Subacute sclerosing panencephalitis (SSPE) was the only other diagnosis that decreased most years after 1994. These decreases appeared to contribute to the overall downward trend in hospitalizations with encephalitis with a known etiology, although this trend was not statistically significant; SSPE hospitalizations declined throughout the 1990s (Figure 5).

## Discussion

Despite being uncommon (5.2/100,000 population annually), encephalitis is of public health importance because rapid response to cases may prevent additional transmitted cases of the underlying etiologies. Currently, nearly 70% of encephalitis cases have no identified etiology, so encephalitis is presently not amenable to public health prevention and control measures. This trend of encephalitis with unidentified etiology is seen elsewhere in developed countries and seems to occur even in the presence of extensive laboratory testing (*22*). Because of the number of emerging infectious diseases associated with encephalitis in Australia and elsewhere in recent times, surveillance should be enhanced.

The encephalitis hospitalization rate and case-fatality rate in NSW are lower than estimates from the United States (rate of 5.2 vs. 7.3/100,000/year, respectively; case-fatality rate of 4.6% vs. 7.4%, respectively); however, these differences may result in part from differences in study methods. The US study included hospitalizations for which encephalitis was the primary, secondary, or other discharge diagnosis, whereas our study included only those cases for which encephalitis was the primary discharge diagnosis. Similarly, a study in the United Kingdom found a hospitalization rate of 1.5 per 100,000 per year. The UK study included only cases of viral encephalitis and thus used a smaller number of ICD codes in the data collection than our study. This selection could account for the lower number of cases, although a true difference cannot be ruled out.

Our findings demonstrated a higher rate of encephalitis admissions that had no identified etiology (69.6%) in NSW than has been found in either the United States (59.5%) or the United Kingdom (60.0%). Although different methods of data extraction and different sample sizes may have contributed to these differences, a true difference in pathogens causing encephalitis could exist among these 3 countries. The high proportion of encephalitis cases with unknown etiology across all 3 countries emphasizes the limits of current diagnostic tools and/or the lack of systematic investigation of encephalitis cases. In Australia, the proportion of encephalitis deaths with no identified etiology increased from 47.0% during 1979–1992 to 57.2% during 1993–2006 (C. Huppatz, unpub. data). Although the datasets for hospital inpatients and for death registry data are not directly comparable, this relative increase in deaths from encephalitis with unknown etiology further emphasizes the need for enhanced routine surveillance.

Our data showed a higher rate of total encephalitis admissions for men (54.3%) compared with women (45.7%); this difference is largely due to a higher rate of toxoplasmosis encephalitis hospitalizations for men, a finding similarly observed in the US hospitalization data (*11*). *Toxoplasma* encephalitis is likely associated with HIV infection in the early 1990s.

The proportion of patients with encephalitis with unidentified etiology did not change during the study period, despite innovations in laboratory testing, particularly increased use of PCR testing after its described use for herpes encephalitis in 1990 (*23*). Interestingly, encephalitis with unknown etiology was not higher in rural areas, where lack of laboratory facilities and access to specialty units may constrain diagnosis. We found a similar rate of known to unknown etiologies for urban and rural hospitals, with the exception of 1 HIV referral center that had a high number of toxoplasmosis encephalitis admissions.

Our study showed that the most common known etiology was herpes virus (12.9%); encephalitis hospitalization rates changed little for patients with this virus during the study period, a finding consistent with the US and UK studies (*11*,*17*).

A decrease in encephalitis admissions due to *Toxoplasma* sp. was observed after a peak occurred for this pathogen in 1993. This decline in toxoplasmosis can be explained by the increasing use of prophylactic treatment for *Toxoplasma gondii* infection in people with HIV infection, a recommendation first widely published in Australia in 1994 (*24*).

The decrease in SSPE, a neurodegenerative disorder caused by the persistence of measles virus in the central nervous system (*25*), reflects the success of immunization in Australia. The median incubation period for the development of SSPE after measles infection is 6–8 years (*25*). Our data show a decline in SSPE admissions after this illness peaked in 1991. This decline most likely resulted from a national measles vaccination campaign in Australia in 1987 (*26*). The surprising increase of SSPE in 2005 can be partly explained by 3 admissions of 1 patient with SSPE. This patient’s recurring hospitalizations were identified during a detailed review of encephalitis patients at the John Hunter Hospital, NSW, Australia (C. Huppatz, unpub. data). The decline in SSPE diagnoses builds on the growing evidence of the success of vaccination in decreasing illness and death from measles and its complications (*26*,*27*).

Several limitations are associated with use of hospital coding data to estimate the impact of disease. During the study period, the ICD coding system changed, and ICD-10 (1998–2007) contained several new codes not present in ICD-9 (1990–1997), including codes for zoster encephalitis, listeria meningoencephalitis, and enteroviral encephalitis. Before 1998, ICD-9 codes must have been designated to conditions associated with the new ICD-10 codes; however, which codes were used cannot definitely be determined. Fortunately, hospitalizations coded with the unknown ICD-9 codes account for a small proportion (<6%) of all encephalitis cases reported (Table).

In addition to limitations due to the ICD coding changes, differences may exist in diagnostic criteria for encephalitis used by clinicians and in inconsistencies among hospitals regarding coding practices and hospital admission criteria. Our data represent hospital admissions, rather than cases of encephalitis. Although this study criterion is useful for estimating the impact of disease as it relates to hospital service provision, case-fatality rates will be underestimated by the use of hospital admission data, rather than true cases, as some patients had multiple hospital admissions. The proportion of encephalitis cases with known and unknown etiologies remained constant, despite the ICD coding system change.

A previous study undertaken in the Northern Territory of Australia also found a high proportion (53%) of encephalitis cases with unknown etiologies (*21*). The Northern Territory has a distinctly different climate from that of NSW, and geographic variation exists in cases of encephalitis with known etiologies. For example, Murray Valley encephalitis and Kunjin encephalitis occur predominantly in the north. The relatively high proportion of encephalitis infections with unknown etiology appears consistent in these 2 different Australian regions.

## Conclusions

Acute encephalitis has heralded the emergence of multiple virulent pathogens (*28*), including West Nile virus, Hendra virus, Nipah virus, Murray Valley encephalitis virus, and Japanese encephalitis virus, all of which can cause severe illness and death (*2*,*4*,*29*–*31*). Although emerging infectious diseases may become apparent due to large outbreaks in humans, such as the Nipah virus outbreak in Malaysia (*4*), the diseases that emerged recently in Australia (e.g., Hendra virus infection and Australian bat lyssavirus infection ) have been found in only a few persons (*2*,*32*). Consistent with US and UK studies (*11*,*17*), we found a high proportion (70%) of encephalitis cases with an unknown etiology in NSW. This finding highlights the need to monitor trends in encephalitis illness in Australia and to improve identification of the etiologies of encephalitis for detection of emerging infectious diseases. Based on our findings, we recommend that in Australia, surveillance be considered for encephalitis to assist in pathogen identification, alert authorities to outbreaks, and allow timely public health action.
